# Mechanism and Basis of Traditional Chinese Medicine Against Obesity: Prevention and Treatment Strategies

**DOI:** 10.3389/fphar.2021.615895

**Published:** 2021-03-08

**Authors:** Chang-hua Zhang, Jun-qing Sheng, Wei-hua Xie, Xiao-quan Luo, Ya-nan Xue, Guo-Liang Xu, Chen Chen

**Affiliations:** ^1^ College of Pharmacy, Jiangxi University of Traditional Chinese Medicine, Nanchang, China; ^2^ College of Life Science, Nanchang University, Nanchang, China; ^3^ Experimental Animal Science and Technology Center of TCM, Jiangxi University of Traditional Chinese Medicine, Nanchang, China; ^4^ Research Center for Differentiation and Development of Basic Theory of TCM, Jiangxi University of Traditional Chinese Medicine, Nanchang, China; ^5^ School of Biomedical Sciences, University of Queensland, Brisbane, QLD, Australia

**Keywords:** traditional Chinese medicine, metabolic disorders, inflammation, dampness-heat syndrome, hypoxia, obesity

## Abstract

In the last few decades, the incidences of obesity and related metabolic disorders worldwide have increased dramatically. Major pathophysiology of obesity is termed “lipotoxicity” in modern western medicine (MWM) or “dampness-heat” in traditional Chinese medicine (TCM). “Dampness-heat” is a very common and critically important syndrome to guild clinical treatment in TCM. However, the pathogenesis of obesity in TCM is not fully clarified, especially by MWM theories compared to TCM. In this review, the mechanism underlying the action of TCM in the treatment of obesity and related metabolic disorders was thoroughly discussed, and prevention and treatment strategies were proposed accordingly. Hypoxia and inflammation caused by lipotoxicity exist in obesity and are key pathophysiological characteristics of “dampness-heat” syndrome in TCM. “Dampness-heat” is prevalent in chronic low-grade systemic inflammation, prone to insulin resistance (IR), and causes variant metabolic disorders. In particular, the MWM theories of hypoxia and inflammation were applied to explain the “dampness-heat” syndrome of TCM, and we summarized and proposed the pathological path of obesity: lipotoxicity, hypoxia or chronic low-grade inflammation, IR, and metabolic disorders. This provides significant enrichment to the scientific connotation of TCM theories and promotes the modernization of TCM.

## Introduction

In the last few decades, the number of cases of obesity and related diseases has significantly increased globally, with more than 1.9 billion overweight adults and 650 million obese adults by 2019 (Ahirwar and Mondal, 2019). The increased prevalence of obesity is related to the increased incidence of metabolic disorders. Obesity is defined as excessive body weight, which is caused by the excessive and unproportioned amount of energy storage as adipose tissues (González-Muniesa et al., 2017; Blüher, 2019; San-Cristobal et al., 2020). Obesity is therefore characterized more particularly by an increase in the quantity of adipose tissue. Such an increase in fat progressively promotes the imbalance of energy storage and expenditure, showing glucose, lipid, and protein metabolic disorders (Charakida et al., 2014). Obesity leads to chronic low-grade inflammation (Bell et al., 2018). In terms of TCM, obese patients have internal “heat” or specifically “dampness heat” (Shi Re in Chinese) with special signs of hot and wet appearance in patients, which is a very important TCM syndrome often observed in obese patients. The development of obesity is closely related to severe metabolic disorders, such as insulin resistance (IR), type 2 diabetes (T2DM), and liver steatosis. These pathological changes cause severe morbidity and mortality in patients (Saltiel and Olefsky, 2017; Ghaben and Scherer, 2019; Yu et al., 2019) and create a significant burden for individuals, families, and society. Today, eating habits and lifestyles have markedly changed in the population (Malik et al., 2013), and a sedentary lifestyle reduces energy consumption (Guthold et al., 2018; San-Cristobal et al., 2020). In TCM, the diagnosis and treatment of diseases are based on a method of differentiation of signs and symptoms known as “syndrome” differentiation or “ZHENG”, which relies on the gathering of clinical information through inspection, auscultation and olfaction, inquiry, and palpation (Chen et al., 2012). One of the most common “ZHENG” in TCM is “dampness-heat syndrome” (DHS) (Shi Re Zheng) (Dai et al., 2013; Yao et al., 2017), which is thought to be caused by a combination of “dampness” and “heat”. DHS can be caused by either external or internal sources (Dai et al., 2013). In addition, hypoxia is also an important feature occurring in this DHS (Lu, 2010).

DHS is prevalent in obesity with chronic low-grade systemic inflammation, is prone to IR, and eventually leads to severe obese metabolic disorders. In the following parts of this review, we focus on the mechanisms underlying obesity with DHS in TCM and propose potential prevention and treatment strategies using TCM theory. To fulfill the purpose, the current knowledge of modern western medicine (MWM) is applied to explain clearly DHS identified by the classic theories of TCM. Hopefully, it may enrich the scientific connotation of TCM theories and promote the modernization of TCM.

## The Relationship Between Adipose Tissue, DHS and Metabolic Disorders

The TCM description of obesity was first seen in the ancient book “Nei Jing”. An unhealthy diet was considered an important cause of obesity. “Su Wen • Qi Bing Lun” described how “The people enjoyed rich food and became fat, experiencing internal heat and dampness. Fat made people hot, sweet made people full.” Sitting for a long period and lack of exercise were also important causes of obesity (Mao, 2005). It was clearly pointed out that partial edible fat and thick oily or sweet taste food with obesity were the causes of the DHS, while internal heat due to fat accumulation and unhealthy food was generated to account for the pathogenesis of DHS (Guo et al., 2020).

In a state of overnutrition, excessive calories need to be stored in adipose tissue. Adipose tissue maintains proper blood vessel formation, insulin sensitivity, and the levels of the anti-inflammatory hormone adiponectin and other metabolic regulatory adipokines. However, progressive adipocyte hypertrophy leads to increased hypoxia with relatively insufficient blood circulation in adipose tissue, which leads to tissue fibrosis with insufficient blood vessel formation. Moreover, hypoxic fat cells are apoptotic and necrotic, leading to immune cell infiltration and tissue inflammation. These factors together lead to a decrease in adipose tissue function, an increase in blood glucose and lipid levels, and lipid deposition in non-fat tissues such as muscle and liver tissues (Ghaben and Scherer, 2019), which is termed “lipid-toxicity (Fei Du in TCM)”.

Nowadays, people generally have more than enough food supply. A normal day-to-day diet is usually above the threshold of required nutrition with excessive fatty and greasy food. The amount of exercise decreases with reduced energy expenditure. Accumulated excessive energy causes an increase in fat tissues, showing a symptom of internal humid or “dampness” and internal hot or “heat” in TCM (Tian et al., 2018). The word “dampness-heat (Shi Re)” first appeared in the “Huang Di Nei Jing”, which was considered to be the collective name of “wet evil” (Shi Xie) and “hot evil” (Re Xie). The book “Zheng Yin Mai Zhi” argues as follows: “Or the order of hot and humid ... The moisture stays for a long period, and becomes hot”. DHS refers to the syndrome related to “dampness-heat” in various clinical diseases (Guo et al., 2020). DHS exhibits the main pathological features of body internal retention of excess water or moisture without “polymerization” and internal accumulation of dampness-heat without “transpiration” (Zhang et al., 2011). The clinical manifestations are chest distress, accumulated internal heat without volatilization, tiredness, heavy and soft limbs, dripping and burning yellow urine, dry stool, and a thick yellow tongue coating (Li et al., 2020). DHS is often a chronic disease that is difficult to cure. In MWM, such chronic disease may include metabolic disorders, type 2 diabetes, obesity, etc. (Tian et al., 2018). DHS was often observed in diabetes, fatty liver, obesity (Xiang et al., 2018), and usually severe IR (Yin and Wei, 2012). The fatty liver was caused by excessive fatty food and the accumulation of evil energy “Qi” in the liver (Liu, 2019). Diabetes belongs to the category of weight-loss and thirsty or “Xiao Ke” in TCM. The most obvious feature is thirsty but no-intention to drink or thirsty without drinking much water. In TCM, it may be caused by the combination of dampness and heat as “dampness-heat” (Huang, 2014). Releasing the heat and removing dampness are the principles in the treatment of DHS (Tian et al., 2018; Li et al., 2020).

## The Relationship Between Hypoxia and Obesity

**FIGURE 1:**
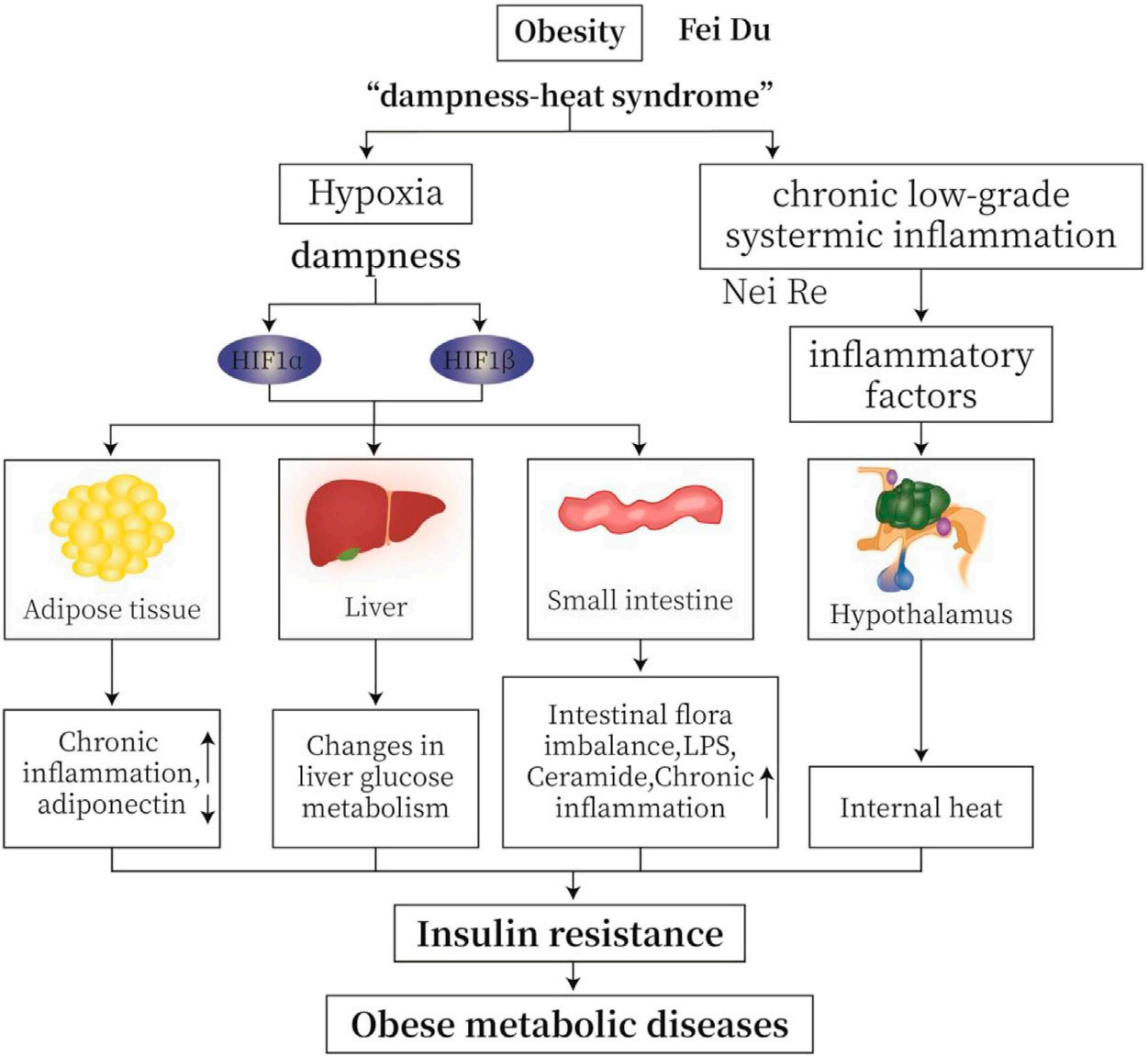
TCM (traditional Chinese medicine) “dampness-heat syndrome” and MWM (modern western medicine) obesity with hypoxia and inflammation. “Dampness” causes intracellular and extracellular hypoxia in adipose tissue and small intestine. Hypoxia is an important feature of “dampness syndrome”. Chronic low-grade inflammation is common in obese patients. From MWM analysis, inflammation may cause temperature or “heat”. Hypoxia induces the production of HIFs, leading to increased production of inflammatory factors, decreased secretion of adiponectin, altered glucose metabolism, imbalanced intestinal flora, and increased lipopolysaccharide (LPS). All these changes cause insulin resistance (IR), and ultimately obese metabolic diseases.

In TCM, obesity belongs to the “dampness-heat syndrome”. “Dampness” causes disturbance of circulation and hypoxia in adipose tissue and small intestine. Hypoxia is an important feature of “dampness” (Lu, 2010). Chronic low-grade inflammation is common in obese patients. In MWM, inflammation causes temperature raise or “heat”. Hypoxia increases the production of hypoxia-inducible factors (HIFs), leading to an increase in inflammatory factors, a decrease in adiponectin, a disturbance of glucose metabolism, imbalance of intestinal flora, and an increase in lipopolysaccharide (LPS); all factors together cause chronic inflammation, IR, and obese metabolic diseases (Figure 1).

Obesity triggers hypoxia in adipose tissue and small intestine, leading to adverse metabolic effects, including IR and non-alcoholic fatty liver disease (NAFLD) (Gonzalez et al., 2018). With the increase in adipose tissue, inflammation and hypoxia occur in adipose tissue to cause insulin resistance (Ichiki and Sunagawa, 2014; Lin and Yun, 2015). When adipocyte size increases, the oxygen supply decreases due to the decrease in capillary density. The increased consumption of oxygen by fat cells in obesity triggers the expression of HIF-1α, leading to inflammation and IR (Ban et al., 2014; Lee et al., 2014). Hypoxia stimulates the secretion of many inflammation-related adipokines (Wood et al., 2009; Trayhurn, 2013) and inhibits adiponectin release from fat tissues of obese people (Ye et al., 2007). Due to a combination of accumulated saturated fatty acids and expression of ADP/ATP translocase 2 (ANT2) in the mitochondria, adipose tissue becomes hypoxic with an increase in uncoupled respiration in the mitochondria (Gonzalez et al., 2018). This decoupling leads to the stabilization of HIF1α. HIF1α induces the expression of cytokine signaling inhibitor 3 (SOCS3), activates Janus kinase (JAK) to phosphorylate SOCS3, activates transcription 3 (STAT3), and inhibits the expression of adiponectin (Gonzalez et al., 2018). Here, hypoxia also increases the instability of adiponectin mRNA (Hosogai et al., 2007). In addition, hypoxia inhibits the differentiation of adipocytes; such inhibition is conducive to the expansion of adipose tissue (Arias-Loste et al., 2015). Moreover, hypoxia increases the expression of genes related to fat formation (Piguet et al., 2009), induces IR in adipose tissue, and promotes fibrosis of adipose tissue (Wood et al., 2009; Trayhurn, 2013).

In obesity, the small intestine becomes hypoxic, resulting in the accumulation of hypoxia HIF2α in the intestine epithelial cells. HIF2α stimulates the expression of gene encoding sialidase 3 (NEU3), which hydrolyzes gangliosides to form ceramides. Elevated ceramide levels lead to obesity due to decreased fat browning, increased fatty acid synthesis, and IR (Gonzalez et al., 2018). Chronic activation of HIF2α leads to increased inflammation and fibrosis and decreased fatty acid β-oxidation. These changes adversely affect liver physiology, leading to NAFLD and non-alcoholic steatohepatitis (NASH) (Gonzalez et al., 2018). Stable liver HIF-2β improves insulin sensitivity (Taniguchi et al., 2013).

Under the hypoxic condition, a rapid increase in the expression of pro-inflammatory cytokines and fibro-genic genes is observed (Qu et al., 2011). Hypoxia induces inflammation in adipose tissue by regulating gene expression in adipocytes and macrophages. The inflammation-related genes include genes encoding TNF-α, IL-1, IL-6, etc. (Semenza et al., 2000; Ye, 2009).

HIFs are the main regulators of hypoxia adaptation and inflammation. HIFs contribute to inflammation through action on cells involved in innate immunity (Imtiyaz and Simon, 2010). HIFs are a family of transcription factors activated by hypoxia and consist of one α subunit (HIF1α, HIF2α or HIF3α) and one β subunit (HIF1β) (Zhang et al., 2010; Klöting and Blüher, 2014).

**FIGURE 2:**
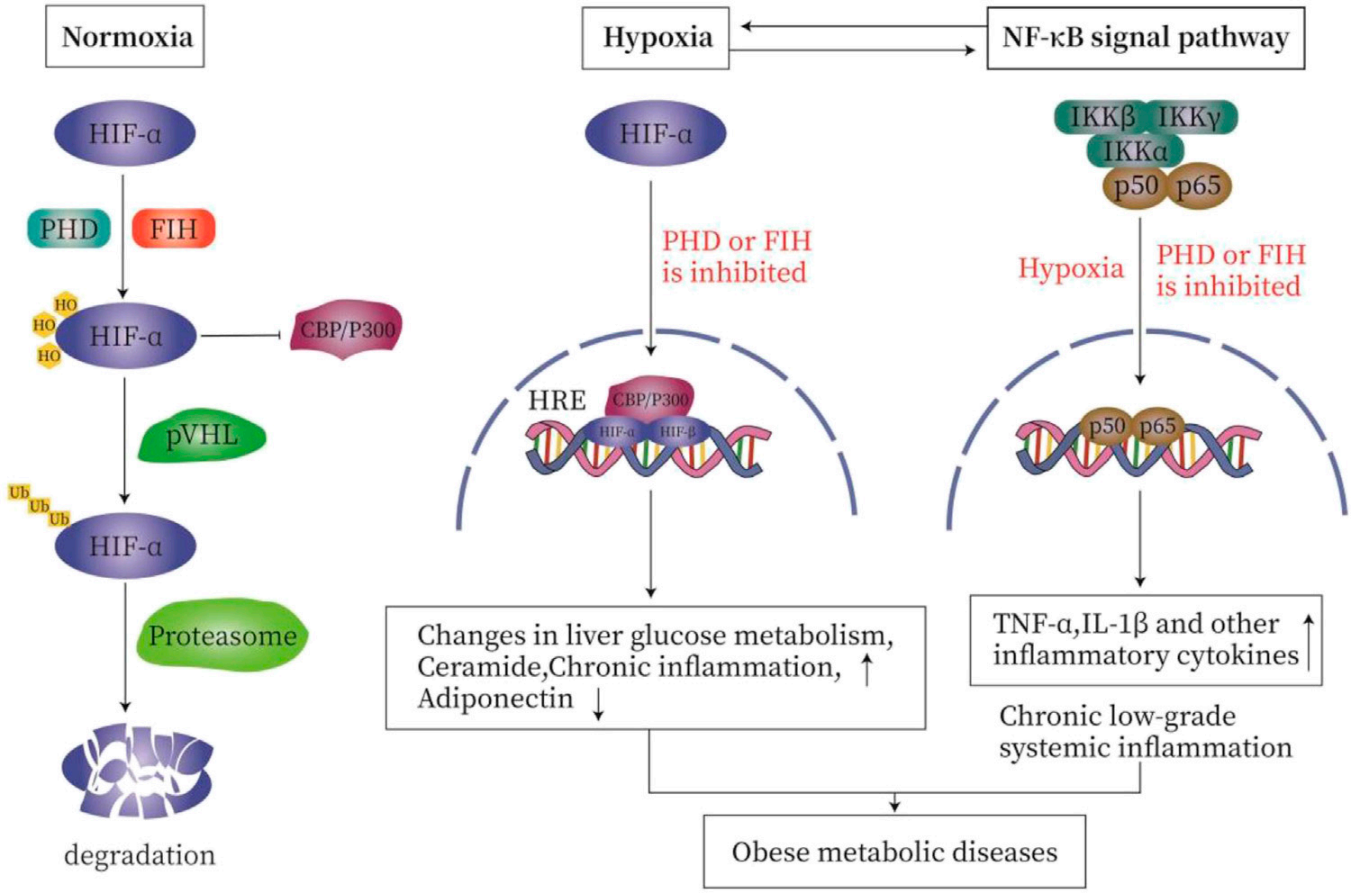
The relationship between hypoxia and inflammation. Under normal oxygen **(A)**, the proline residues on the α subunit of HIF are hydroxylated by HIF prolyl hydroxylase, so that HIF is recognized and ubiquitinated by VHL E3 ubiquitin ligase. It is then rapidly degraded by the proteasome. Asparagine hydroxylation inhibits the interaction of HIFα with the co-activator cAMP response element binding (CREB)-binding protein (CBP) and histone acetyltransferase p300 (p300). During hypoxia **(B)**, the enzyme activities of PHD and FIH are inhibited, resulting in the stabilization of HIFα subunit. After being transported to the nucleus, HIFα complexes with the β subunit, recruits p300 and CBP, and then binds to the hypoxia response element (HRE) in the target gene promoter to initiate transcription. Hypoxia and inflammation **(C)**. On the one hand, inflammation is usually characterized by tissue hypoxia or the stabilization of hypoxia-dependent transcription factors such as hypoxia-inducible factor (HIF). On the other hand, hypoxia is characterized by secondary inflammatory changes. In order to meet the challenge of hypoxia and ensure cell survival, the regulation of HIF and nuclear factor-κB (NF-κB) occurs through oxygen-sensitive prolyl hydroxylase (PHD). Hypoxia activates NF-κB through a pathway involving IκB kinase-β (IKKβ) activation, which leads to phosphorylation-dependent degradation of IκBα and the release of NF-κB. PHD and FIH regulate the activation of NF-κB by regulating the activity of IKKβ.

Under normoxia, the proline residues on the α subunit of HIF are hydroxylated by oxygen-sensitive prolyl hydroxylase (PHD) so that the HIF is recognized and ubiquitinated by VHL E3 ubiquitin ligase and rapidly degraded by the proteasome. The asparagine hydroxylation inhibits the interaction of HIFα with the co-activators cAMP response element binding (CREB), cAMP binding protein (CBP), and histone acetyltransferase p300 (p300). During hypoxia, the enzyme activities of PHD is reduced, resulting in the stabilization of the HIFα subunit. After being transported to the nucleus, HIFα subunit complex with the β subunits recruits p300 and CBP, and binds to the hypoxia response element (HRE) to promote the target gene to initiate specific transcription (Arias-Loste et al., 2015; Lefere et al., 2016; Taylor and Colgan, 2017; Van Welden et al., 2017; Gonzalez et al., 2018) (Figure 2).

Hypoxia and inflammation are related and interact with each other. On the one hand, inflammatory diseases are usually characterized by tissue hypoxia or the stabilization of hypoxia-dependent transcription factors such as HIF. On the other hand, hypoxia-caused diseases are characterized by secondary inflammatory changes (Bartels et al., 2013). In order to meet the challenge of hypoxia and to ensure cell survival, the HIF and nuclear factor-κB (NF-κB) are activated; both are regulated by oxygen-sensitive prolyl hydroxylase (PHD) (Van Welden et al., 2017). In addition to HIF-1α, NF-kB is also activated by hypoxia (Ye et al., 2007). Hypoxia activates NF-κB through a pathway involving IκB kinase-β (IKKβ) activation, which leads to phosphorylation-dependent degradation of IκBα and the release of NF-κB (Michiels et al., 2002; Bartels et al., 2013). PHD and FIH regulate the activation of NF-κB by controlling the activity of IKKβ (Eltzschig and Carmeliet, 2011). Hypoxia induces IKKβ activation by inhibiting PHD activities (Devraj et al., 2017). PHD2 hydroxylates IKKβ, while PHD3 prevents the interactions between IKKβ and heat shock protein 90 (HSP90) and between IKKγ and apoptosis inhibitor (cIAP1). PHD1 may hydroxylate IKKβ, but it has not been fully established (Van Welden et al., 2017) (Figure 2).

Hypoxia promotes changes in mitochondrial structure and genome stability, resulting in reduced mitochondrial respiration, reduced ATP production, and accumulated mtDNA mutation (Vega et al., 2015). MtDNA-mediated inflammation is driven by the activation of inflammasomes (McGarry et al., 2018). The NOD-like receptor family protein 3 (NLRP3) inflammasome is a target of mtDNA; activation of NLRP3 leads to the subsequent activation of caspase-1 and the secretion of IL-1β and IL-18 (Nakahira et al., 2011). IL-1β belongs to the family of interleukin-1 cytokines and is activated by NF-kB-mediated HIF-1α (Jung et al., 2003). Hypoxic conditions increase levels of reactive oxygen species (ROS) and oxidative stress (McGarry et al., 2018); here, mitochondrial ROS may induce the activation of NLRP3 inflammasome (Zhou et al., 2011).

In addition to being induced by hypoxia, other non-hypoxic stimuli, such as lipopolysaccharide (LPS) and pro-inflammatory cytokines, may induce NF-κB- dependent increase in HIF1 mRNA levels. HIF promotes the activation of NF-κB (Van Welden et al., 2017). LPS is the main component of Gram-negative bacterial membranes and activates HIF-1 in macrophages or monocytes (Devraj et al., 2017). In macrophages, LPS regulates the activation of hypoxia-regulated genes through the HIF-1 pathway (Blouin et al., 2004). LPS is recognized by Toll-like receptors (TLR) expressed on myeloid cells. The downstream signal transduction of TLR involves NF-κB, which increases the expression of HIF-1 and key inflammatory cytokines such as tumor necrosis factor α (Arias-Loste et al., 2015). In addition, certain types of intestinal flora are responsible for the production of short-chain fatty acids (SCFA) (acetic acid, propionic acid, and butyric acid). The intestinal epithelial cells at the top of the villi are the main users of butyric acid. Peroxisome proliferation PPARγ (inducible by butyrate) in the cells activates β-oxidation and oxidative phosphorylation. In this process, a large amount of oxygen is consumed, which makes the top of the intestinal crypts physiologically hypoxic (Maslowski, 2019).

## The Relationship Between Chronic Low-Grade Inflammation, Insulin Resistance, and Metabolic Disorders

“Dampness-heat” is closely related to inflammatory factors (Guo et al., 2020). DHS is mostly reflected by the changes in inflammatory factors and abnormal immune function. It is also closely related to oxidative damage, energy metabolism, endotoxin, blood lipid metabolism, etc. (Tian et al., 2018).

The inflammatory response plays an important role in the pathogenesis of obesity and related chronic diseases (Rodríguez-Hernández et al., 2013). There is strong evidence that obesity is closely associated with chronic low-grade systemic inflammation, which is a key element to the occurrence and development of obese metabolic disorders (Crunkhorn, 2013; Lasselin and Capuron, 2014; Yu et al., 2019). Obesity- and inflammation-related metabolic disorders usually show clear IR (Sun and Karin, 2012).

Chronic low-grade inflammation has no signs of concomitant infection or autoimmunity and no large-scale tissue damage. In addition, the inflammation activation is often limited and referred to as “low-grade” chronic inflammation (Monteiro and Azevedo, 2010). Obesity-related chronic low-grade inflammation has the activation of various inflammatory signaling cascades leading to the activation of NF-κB, Jun N-terminal kinase (JNK), and inflammatory bodies (Catrysse and van Loo, 2017). Chronic inflammation with metabolic syndrome is the inflammation in multiple organs and tissues, including adipose tissue, pancreas, liver, muscle, hypothalamus, and gastrointestinal tract (Yu et al., 2019).

Adipose tissue inflammation is considered a key event leading to metabolic diseases (Yu et al., 2019). In dysfunctional hypertrophic adipose tissue, lipolysis increases to cause excessive free fatty acid (FFA) production. This leads to mitochondrial dysfunction and oxidative stress in adipose tissue and activates the inflammatory response through NF-κB. FFAs indirectly bind to Toll-like receptor (TLR) 4 and TLR2 through the adaptor protein fetuin A, thereby promoting inflammation and activation of NF-κB and JNK. Once activated, NF-κB and JNK pathways increase the synthesis and secretion of inflammatory factors and chemokines in adipocytes and hepatocytes (Shi et al., 2006; Calle and Fernandez, 2012; Saltiel and Olefsky, 2017). In addition, excessive nutrients may overload the function of the endoplasmic reticulum, and lead to more protein misfolding by activating the unfolded protein reaction (UPR). UPR induces activation of NF-κB through transcription factor 6 and other factors to promote the pro-inflammatory response and contributes to low-grade inflammation in obesity (Hotamisligil and Erbay, 2008; Calle and Fernandez, 2012). Adipose tissue is a heterogeneous mixture of adipocytes, interstitial preadipocytes, immune cells, and the endothelium. In obesity, with the increase in the size of adipocytes due to lipid accumulation, the blood supply to adipocytes decreases relatively to cause hypoxia. Hypoxia triggers macrophage infiltration, leading to excessive production of pro-inflammatory factors to disturb the insulin receptor signaling cascade and to cause IR. In addition, hypoxia induces inflammation of macrophages and inhibits the differentiation of preadipocytes (Hotamisligil and Erbay, 2008; Calle and Fernandez, 2012; Emanuela et al., 2012). Obesity induces adipocyte hypertrophy and changes the composition of epidermal vascular cells into a pro-inflammatory state. In lean adipose tissue, type 2 T helper cells and regulatory T lymphocytes promote the polarization of M2 macrophages, thereby maintaining an anti-inflammatory state. In obesity, adipose tissue is characterized by the enrichment of macrophages and T lymphocytes, changing from an anti-inflammatory state to a pro-inflammatory state. Cytotoxic CD8 +, type 1 T helper cells, and type 17 T helper cells stimulate the polarization of M1 macrophages. In obesity, the imbalance between different immune cells leads to the excessive production of chemokines and pro-inflammatory cytokines, which then promote systemic inflammation and peripheral tissue IR (Esser et al., 2014; Saltiel and Olefsky, 2017). M1-like macrophages secrete inflammatory cytokines, which induce IR locally or enter the circulation to cause systemic IR and inflammation (McNelis and Olefsky, 2014). Cytokines secreted locally by M1 proinflammatory adipose tissue macrophages (such as TNF-α) initiate the NF-κB and JNK pathways. Reduced production of ceramide prevents IR induced by saturated fatty acids (Saltiel and Olefsky, 2017). In addition, adiponectin and PPARγ directly polarized mouse macrophages or human monocytes to anti-inflammatory M2 macrophages (Fuentes et al., 2013). Inflammation in the pancreatic islets led to β-cell dysfunction and apoptosis, resulting in insufficient insulin secretion (Eguchi and Nagai, 2017). Hepatic steatosis is a common feature of obesity, which develops to NAFLD and eventually NASH and fibrosis. Two main types of macrophages were found in the liver, Kupffer cells (KC) and recruited hepatic macrophages (RHM). KC is a resident macrophage and serving as sentinels for liver homeostasis (Tacke, 2017). RHM accumulates in large amounts in the liver and is highly proinflammatory (Li et al., 2015). In obesity, many pro-inflammatory immune cells (including M1 macrophages) infiltrated and accumulated in muscle tissue, which secreted pro-inflammatory cytokines, such as TNF-α, and IL-1β (Esseretal et al., 2014; Khan et al., 2015). In addition, obesity was also related to changes in brain function, especially in the hypothalamus that regulates energy homeostasis and systemic metabolism (Saltiel and Olefsky, 2017). Clinical and experimental studies demonstrated that resistin was a key hormone that linked hypothalamic inflammation and IR in obesity through the TLR4 signaling pathway (Jais and Brüning, 2017). The gastrointestinal microbiota may produce metabolites and by-products to induce proinflammatory cytokines and regulate metabolism through the development of obesity (Yu et al., 2019). Changes in the microbiome led to intestinal mucosal barrier damage with increased endotoxin absorption to promote inflammation and fibrosis through TLR signal transduction (Bieghs and Trautwein, 2013).

**FIGURE 3:**
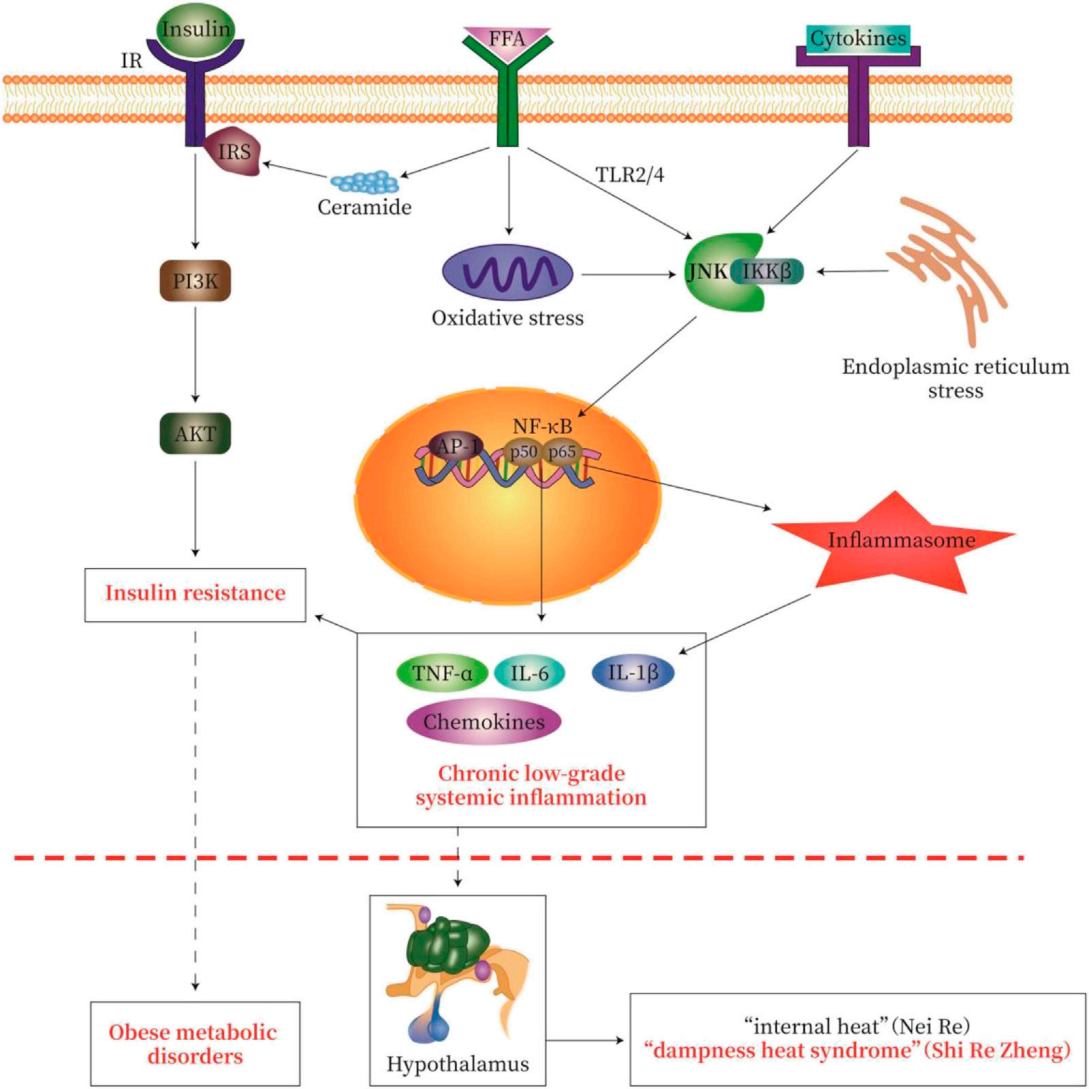
Pathological pathway of “Lipid-toxicity Inflammation” or “Dampness-heat Syndrome”—Insulin Resistance-Metabolic disease. We try to explain the classic theory of TCM by using modern western medicine (MWM). In obese patients with dampness heat syndrome, a large amount of free fatty acid (FFA) is produced in the body, causing lipotoxicity, called “lipid toxicity or Fei Du”. A large amount of FFA may cause chronic low-grade systemic inflammation, which causes “heat” as one symptom of inflammation. These may be the modern medical interpretation of “fat makes people hot” in dampness heat syndrome. The severity of obesity-type metabolic disorders (diabetes, fatty liver, obesity) reflects the degree of inflammatory injury. The activation of various inflammatory signaling cascades in chronic low-grade systemic inflammation leads to the activation of NF-κB, Jun N-terminal kinase (JNK), and inflammatory bodies, thereby causing insulin resistance. The common pathological feature of obese metabolic disorders is chronic low-grade inflammation and insulin resistance. IR, insulin receptor; IRS, insulin receptor substrate; FFA, free fatty acids. The signal pathway map here was generated by Adobe Illustrator software.

Activation of TLR2, TLR4, and/or tumor necrosis factor receptor (TNFR) leads to activation of NF-κB and JNK signaling. Serine kinases IKKβ and JNK phosphorylate serine residues of IRS-1 and IRS-2 and inhibit downstream insulin signaling to cause IR. In addition, the activation of IKKβ causes NF-κB to translocate to the nucleus. Similarly, activation of JNK leads to the formation of activator protein-1 (AP-1) transcription factors. Nuclear NF-κB and AP-1 activate inflammatory genes, which may promote further IR (McNelis and Olefsky, 2014; Catrysse and van Loo, 2017; Zhang et al., 2020). In addition, TLR signaling increases cellular responsiveness to stimuli derived by the assembly and activity of inflammatory molecules; such an increase in responsiveness effectively enhances the efficiency of inflammatory molecule assembly, the release of IL-1β (Fitzgerald and Kagan, 2020), and IR (Figure 3).

In addition, short-chain fatty acids (SCFAs) and the farnesoid X receptor (FXR) are key targets closely related to obesity-related glucose metabolism, lipid metabolism, and microbial metabolism. SCFAs are the important molecules linking glycolipid metabolism to intestinal microbial metabolism, and the metabolites of intestinal microbial metabolism (Chassaing et al., 2015). SCFAs are key factors regulating lipid metabolism, including acetic acid, propionic acid, butyric acid, etc (Koh et al., 2016). SCFAs therefore play an important role in regulating host metabolism, mediating inflammatory processes, and maintaining energy homeostasis (Sanchez et al., 2020).

Moreover, FXR is a key receptor regulating intestinal microbes and glycolipid metabolism. FXR is a transcription factor of the nuclear hormones activated by bile salts. Bile acid (BA) is an endocrine molecule that not only promotes the absorption of fat-soluble nutrients but also regulates many metabolic processes, including glucose, lipid, and energy homeostasis (Molinaro et al., 2018). BA and metabolites of BA by the action of intestinal flora may activate FXR (Schubert et al., 2017; Marra and Svegliati-Baroni, 2018). FXR activation may promote the action of fibroblast growth factor 15/19 (FGF-15/19) and cholesterol-7α-hydroxylase (CYP7A1), to regulate TG metabolism and gluconeogenesis (Jones et al., 2014; Marra and Svegliati-Baroni, 2018).

## Prevention and Treatment Strategies

Obesity and related metabolic disorders are a global epidemic, leading to increased mortality and medical costs. No effective treatment options have been established (Dai et al., 2013; Saltiel and Olefsky, 2017; Ghaben and Scherer, 2019). The best way nowadays is to change eating habits and lifestyle and to participate actively in physical exercise. The incidence of T2DM is equally increasing, and Asia populations are particularly prevalent in T2DM globally, especially in China and India (Zheng et al., 2018). NAFLD is currently a common metabolic disease. Recently, NAFLD has also been named metabolic-related fatty liver disease (MAFLD) (Eslam et al., 2020), which is characterized by excessive deposition of lipids in the liver. The high incidence of NAFLD affects approximately 25% of the adult population worldwide (Diehl and Day, 2017; Friedman et al., 2018; Paul and Davis, 2018; Eslam et al., 2020). NAFLD may further develop into NASH and eventually liver cancer. These diseases are closely related to obesity and obese metabolic disorders and are often accompanied by chronic low-grade systemic inflammation, identified as obese metabolic disorders in MWM or “dampness-heat” in TCM. Dampness-heat is well established TCM syndrome for obese metabolic disorders.

**FIGURE 4:**
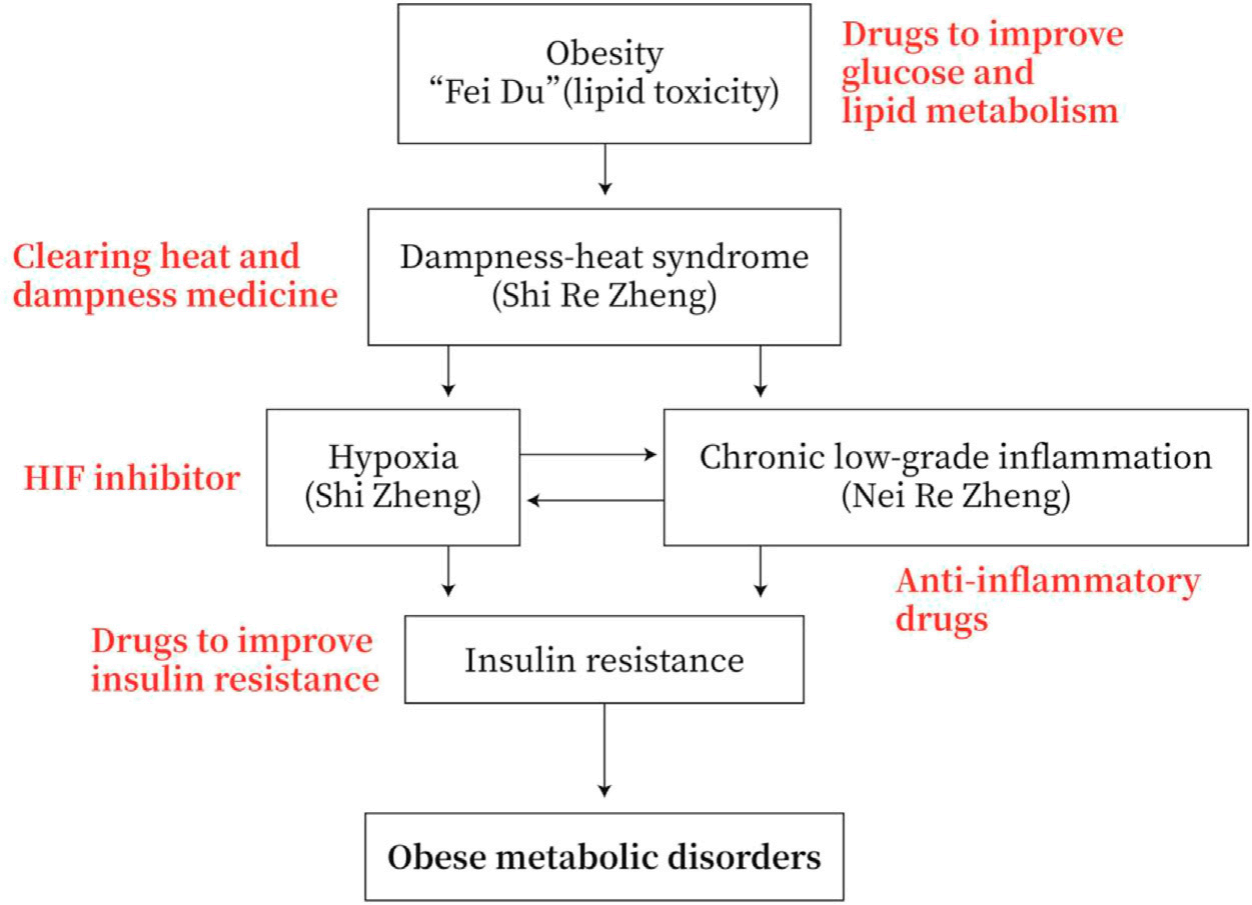
Strategies for the prevention and treatment of obese metabolic disorders. Obesity has the symptoms of “dampness heat”. Obesity patients exhibit dysfunction of glucose and lipid metabolism. They generally have adipose tissue and/or small intestine hypoxia and chronic low-grade inflammation, and they are prone to insulin resistance (IR) and obese metabolic diseases. Therefore, the TCM drugs to prevent and treat obesity-type metabolic diseases are mainly heat-clearing and anti-dampness, or in MWM anti-inflammation, inhibiting HIF and improving IR.

Obesity has the symptoms of “dampness-heat”, with disturbance of glucose and lipid metabolism. Hypoxia is often occurring in adipose tissue and small intestine with chronic low-grade inflammation. These hypoxia and inflammation are prone to IR and eventually lead to obese metabolic diseases. The TCM mainly uses “heat-releasing” and “dampness-cleaning” drugs to prevent and treat obese metabolic diseases or to improve glucose and lipid metabolism, to reduce inflammation, to inhibit HIF, and to reverse IR in MWM (Figure 4).

In MWM, chemical drugs used to prevent obese metabolic diseases mainly include drugs improving metabolism (such as PPARα/δ agonists, PPARγ agonists), anti-inflammation, antioxidants, anti-FXR, regulating intestinal flora, inhibiting DAG and ceramide production, and reducing gluconeogenesis and glycogenolysis (such as metformin), etc. (Zhang et al., 2020). Obesity patients generally have excessive adipose tissue and small intestine hypoxia, chronic low-grade inflammation, and IR, which eventually leads to obese metabolic diseases. Therefore, HIF inhibitors may be used to prevent and treat obese metabolic diseases. Based on the HIF1α-SOCS3-STAT3-adiponectin pathway in adipose tissue, the HIF inhibitor pyridine prevents obesity and IR caused by a high-fat diet (HFD) (Jiang et al., 2013). PX-478 selectively inhibits fat HIF1α, thereby partially improves metabolic dysfunction by reducing fat fibrosis (Sun et al., 2013). In addition, the HIF inhibitor PT2385 reduces the level of ceramide in the intestine and serum, which thereby improves metabolism (Gonzalez et al., 2018).

TCM may act on multiple targets of the pathological pathway of “lipid toxicity (Fei Du)-Inflammation-DHS-IR-Metabolic disease”. TCM drugs, such as medicines for clearing away heat and dampness, medicines for improving glycolipid metabolism, medicines for anti-inflammation, and medicines for improving IR, may be used to prevent and treat obese metabolic disorders (Figure 4).

In this review, Gegen Qinlian Decoction (GGQLD) (prescription), Scutellaria-coptis herb couple (drug pair), Scutellaria baicalensis Georgi or Coptis chinensis Franch (medicinal material), and Baicalin or Berberine (component) have been taken as an example.

First, the influence of traditional Chinese medicine-GGQLD on obesity-related glucose metabolism, lipid metabolism, and microbial metabolism was reported (Zhang et al., 2013; Zhang et al., 2017; Zhang et al., 2020). GGQLD is a classic recipe that originated from Zhang Zhongjing’s “Treatise on Febrile Diseases” to treat DHS (Guo et al., 2020). GGQLD is made up of four herbs: *Pueraria montana var. lobata* (Gegen), *Scutellaria baicalensis Georgi* (Huangqin), *Coptis chinensis Franch* (Huanglian), and *Glycyrrhiza uralensis Fisch*. ex DC (Gancao) (Zhang et al., 2013; Zhang et al., 2020). Based on the analysis of pathogenesis in MWM, GGQLD prevented and treated obese metabolic disorders and improved T2DM and NASH (Zhang et al., 2013; Wang et al., 2015; Guo et al., 2017; Xu et al., 2020; Zhang et al., 2020). Effects of GGQLD were related to the regulation of glucose and lipid metabolism (Zhang et al., 2013; Sui et al., 2019; Tu et al., 2020), intestinal flora (Zhang et al., 2017; Guo et al., 2018; Liu et al., 2019), levels of SCFAs (Liu et al., 2019), and oxidant stress and inflammatory (Zhang et al., 2020). In TCM, Scutellaria-coptis herb couple (SC) in the “Treatise on Febrile Diseases” had the effect of clearing away heat and dampness, purging fire, and detoxifying. The “Compendium of Materia Medica” recorded that “Scutellaria baicalensis Georgi is tasting bitter and causing internal cold, and cures dampness and heat ... the effect is similar to that of Coptis chinensis” (Tian et al., 2018). SC was reported to improve IR in T2DM (Liu et al., 2013; Zhang et al., 2019). In mice with IR induced by high-fat diet, Scutellaria baicalensis Georgi improved IR by inhibiting macrophage-mediated inflammation (Na and Lee 2019). In addition, the extract of Scutellaria baicalensis Georgi showed strong anti-obesity and anti-triglyceride effects (Song et al., 2013), which prevented FFA-induced lipotoxicity through AMPK-mediated SREBP signaling pathway, thereby alleviated NAFLD (Chen et al., 2018). Long-term treatment with baicalin improved diet-induced obesity and hepatic steatosis and led to systemic improvements in many metabolic diseases (Dai et al., 2018). The highest reported dose of baicalin at 400 mg/kg/d was safe without significant side effects and significantly reduced obesity and fatty liver disease (Xi et al., 2015). Moreover, baicalin reduced NASH by inhibiting lipid metabolism, inflammation, and fibrosis in mice (Zhang et al., 2018) and reduced diet-induced NASH by inhibiting the activation of the JNK signaling pathway (Zhong and Liu, 2018). Baicalin was effectively used to treat abnormal blood sugar and blood lipid metabolism caused by a long-term high-fat diet by adjusting the abundance of the microbiota and changing the production of a variety of SCFAs (Ju et al., 2019). Berberine regulates liver lipid metabolism by changing microbial bile acid metabolism and intestinal FXR signaling pathway (Sun et al., 2017).

## Conclusion and Perspectives

Chronic inflammation caused by obesity promotes the development of many metabolic disorders, especially IR, T2DM, and NAFLD (Yu et al., 2019). DHS in TCM is closely related to obesity in MWM, with excessive fat-induced “internal heat and dampness”. In obese patients with DHS, an excessive amount of FFA causes lipotoxicity or “Fei Du” in TCM. This excessive amount of FFA as “Fei Du” causes chronic low-grade systemic inflammation, which evokes internal “heat (Re)” as one of the symptoms of inflammation. The description above reflects the effort to employ modern western medical interpretation of “fat makes people hot”. The severity of obesity-type metabolic disorders with DHS reflects the degree of inflammatory injury. The activation of various inflammatory signaling cascades in chronic low-grade systemic inflammation leads to the activation of NF-κB, JNK, and inflammatory bodies, thereby causing IR (shown in Figure 3). Hypoxia and inflammation are important characteristics of “dampness-heat syndrome”, which is prevalent in chronic low-grade systemic inflammation with IR, and eventually obese metabolic disorders. Chinese medicines act on multiple targets of the pathological pathway of metabolic disorders. These medicines effectively clear internal heat and dampness, improve glycolipid metabolism and IR, and dampen chronic inflammation.

In summary, this article discussed the mechanisms of TCM underlying obesity and related metabolic disorders and proposed accordingly prevention and treatment strategies. In particular, the MWM theories of hypoxia and inflammation were applied to explain the “dampness-heat” syndrome of TCM, and we summarized and proposed the pathological path of obesity, lipotoxicity, hypoxia or chronic low-grade inflammation, IR, and metabolic disorders. Such discussion provides great significance to enrich the scientific connotation of TCM theories and promotes the modernization of TCM.

## Data Availability

The raw data supporting the conclusions of this article will be made available by the authors, without undue reservation.

## Abbreviations

AP-1, activator protein-1; DHS, dampness-heat syndrome; FFA, free fatty acid; FXR, farnesoid X receptor; GGQLD, Gegen Qinlian Decoction; HIF, hypoxia inducible factor; HRE, hypoxia response element; IR, insulin resistance; JNK, Jun N-terminal kinase; KC, Kupffer cells; LPS, lipopolysaccharide; MWM, modern western medicine; NAFLD, Non-alcoholic fatty liver disease; NASH, non-alcoholic steatohepatitis; NF-κB, nuclear factor-κB; NLRP3, NOD-like receptor family protein 3; PHD, prolyl hydroxylase domain; RHM, recruited liver macrophages; SC, Scutellaria-coptis herb couple; SCFAs, short-chain fatty acids; T2DM, type 2 diabetes; TCM, traditional Chinese medicine; TLR, Toll-like receptor; UPR, unfolded protein reaction.
